# Interface Engineering to Drive High‐Performance MXene/PbS Quantum Dot NIR Photodiode

**DOI:** 10.1002/advs.202307169

**Published:** 2023-12-03

**Authors:** Yunxiang Di, Kun Ba, Yan Chen, Xudong Wang, Mingqing Zhang, Xinning Huang, Yi Long, Mengdi Liu, Shukui Zhang, Weiyi Tang, Zhangcheng Huang, Tie Lin, Hong Shen, Xiangjian Meng, Meikang Han, Qi Liu, Jianlu Wang

**Affiliations:** ^1^ State Key Laboratory of Integrated Chips and Systems Frontier Institute of Chip and System Fudan University Shanghai 200433 China; ^2^ Institute of Optoelectronics Shanghai Frontier Base of Intelligent Optoelectronics and Perception Fudan University Shanghai 200433 China; ^3^ State Key Laboratory of Infrared Physics Shanghai Institute of Technical Physics Chinese Academy of Sciences Shanghai 200083 China; ^4^ Department of Materials Science Fudan University Shanghai 200433 China; ^5^ Hangzhou Institute for Advanced Study University of Chinese Academy of Sciences Hangzhou Zhejiang 310024 China; ^6^ Shanghai Qi Zhi Institute 41st Floor, AI Tower, No. 701 Yunjin Road, Xuhui District Shanghai 200232 China

**Keywords:** infrared photodetector, interface engineering, MXene, PbS colloidal quantum dot, transparent conducting electrode

## Abstract

The realization of a controllable transparent conducting system with selective light transparency is crucial for exploring many of the most intriguing effects in top‐illuminated optoelectronic devices. However, the performance is limited by insufficient electrical conductivity, low work function, and vulnerable interface of traditional transparent conducting materials, such as tin‐doped indium oxide. Here, it is reported that two‐dimensional (2D) titanium carbide (Ti_3_C_2_T*_x_*) MXene film acts as an efficient transparent conducting electrode for the lead sulfide (PbS) colloidal quantum dots (CQDs) photodiode with controllable near infrared transmittance. The solution‐processed interface engineering of MXene and PbS layers remarkably reduces the interface defects of MXene/PbS CQDs and the carrier concentration in the PbS layer. The stable Ti_3_C_2_T*_x_*/PbS CQDs photodiodes give rise to a high specific detectivity of 5.51 × 10^12^ cm W^−1^ Hz^1/2^, a large dynamic response range of 140 dB, and a large bandwidth of 0.76 MHz at 940 nm in the self‐powered state, ranking among the most exceptional in terms of comprehensive performance among reported PbS CQDs photodiodes. In contrast with the traditional photodiode technologies, this efficient and stable approach opens a new horizon to construct widely used infrared photodiodes with CQDs and MXenes.

## Graphical abstract

A novel two‐dimensional titanium carbide (Ti_3_C_2_T*_x_*) MXene film is introduced as an effective transparent conducting electrode in lead sulfide quantum dot photodiodes. Exhibiting exceptional near‐infrared transmittance control, interface engineering enhances performance, yielding high detectivity, extensive dynamic response, and notable bandwidth, thereby advancing infrared photodiode technology.

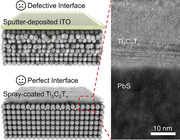

## 1 Introduction

Benefiting from the strong absorption, size‐tunable bandgap, and solution‐processable integration at low temperatures, lead sulfide (PbS) colloidal quantum dots (CQDs) show great promise for cost‐effective photodetectors in the near‐infrared (NIR) and short‐wave‐infrared (SWIR) bands.^[^ ^1^, ^2^, ^3^ ^]^ For PbS CQDs‐based optoelectronic devices, a transparent conducting electrode (TCE) is indispensable for the top‐illumination. Generally, transparent conductive materials, such as tin‐doped indium oxide (ITO),^[^ ^4^ ^]^ carbon nanotubes,^[^ ^5^ ^]^ graphene,^[^ ^6^ ^]^ and conductive polymers,^[^ ^7^ ^]^ are deposited on the top of the devices as a TCE. However, ITO is plagued by brittleness, high cost, and the diffusion of metal species into photosensitive layers.^[^ ^8^ ^]^ The high‐energy particles and plasma emission along with the deposition of ITO film can irreversibly damage the sensitive bottom CQDs layers, which degrade the performance of PbS CQDs photodiodes with active layers.^[^ ^9^ ^]^ To avoid damage, a protective layer, such as fullerene, has been developed to construct a junction with low interface defect density on PbS CQDs layers.^[^ ^10^ ^]^ However, the additional layer increases manufacturing complexity and cost. Ideally, a solution‐processable TCE with high electrical conductivity and selective transparency is more compatible to the solution‐based CQDs photodiode.

MXenes, as an expanding group of 2D transition‐metal carbides, nitrides, or carbonitrides, have the general formula of M_n+1_X_n_T*_x_*, where M, X, and T*_x_* refer to an early transition metal (Ti, V, Nb, Mo, etc.), C and/or N, and surface functional group (‐O, ‐OH, ‐F, ‐Cl, etc.), respectively.^[^ ^11^, ^12^ ^]^ The electrical conductivity of Ti_3_C_2_T*_x_* film reaches 8000−25000 S/cm depending on the quality of MXene and the film manufacturing process.^[^ ^13^ ^]^ The single Ti_3_C_2_T*_x_* layer can transmit ≈97% of visible light.^[^ ^14^ ^]^ Moreover, solution‐processable MXene is compatible with various film techniques such as spray‐coating,^[^ ^15^ ^]^ spin‐coating,^[^ ^16^ ^]^ blade‐coating,^[^ ^17^ ^]^ etc. These features suggest that MXene holds promise as a TCE for photodetectors. For example, an MXene‐GaN‐MXene metal‐semiconductor‐metal photodetector exhibited a reduced dark current and improved specific detectivity compared to traditional Cr/Au‐GaN‐Cr/Au photodetectors.^[^ ^18^ ^]^ However, it is still challenging to develop MXene as the TCE for top‐illuminated PbS CQDs photodetectors, such as selective IR transmittance, insecure environmental stability, and uncertain compatibility with the PbS CQD fabrication process.

Herein, we report the solution‐processed interface design of MXene and PbS layers for top‐illuminated CQD photodiodes. The work function (*WF*) and NIR optical transmittance of spray‐coated MXene films with different thicknesses were investigated. The interface contact was demonstrated through the NIR photodiode device with top‐illuminated Au/ZnO/PbS‐I/PbS‐EDT/MXene. The defect density of Ti_3_C_2_T*_x_*/PbS CQDs NIR photodiode provides insight into the rational interface design. The reliability of the MXene/PbS CQDs NIR photodiode was examined in ambient conditions. The IR imaging capability of the detector in a self‐powered state was validated. This work not only illustrates the suitability of MXene for CQD IR imagers but also provides a realistic approach to developing MXene electrodes for diverse electronic and optoelectronic devices.

## 2 Results and Discussion

**Figure 1:**
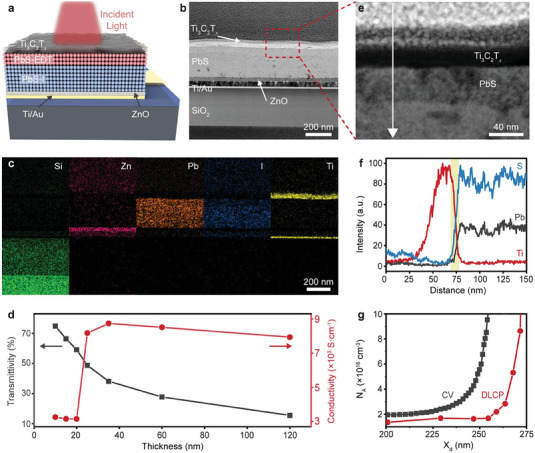
Structural characterization of Ti_3_C_2_T*_x_*/PbS CQDs NIR photodiode. a) Schematic diagram of the Ti_3_C_2_T*_x_*/PbS CQDs NIR photodiode. b) Cross‐section TEM image of the Ti_3_C_2_T*_x_*/PbS CQDs NIR photodiode. c) EDS elemental mapping of Si (green), Au (violet), Zn (pink), Pb (brown), I (blue), and Ti (yellow) of the Ti_3_C_2_T*_x_*/PbS CQDs NIR photodiode. d) Thickness‐dependent electrical conductivity of the spray‐coated Ti_3_C_2_T*_x_* films and the corresponding transmittivity at 940 nm. e) High‐resolution cross‐section TEM image of the Ti_3_C_2_T*_x_*/PbS CQDs interface and f) elemental distribution profiles along the direction indicated by the arrow in (e). g) Defect density profiles of Ti_3_C_2_T*_x_*/PbS CQDs NIR photodiode by *C–V* and DLCP measurement. *X*_d_ is the depletion width of the photodiode.

PbS CQDs‐based photodiodes are generally designed as top‐illuminated structures to enable incident‐light absorption and signal acquisition. A schematic diagram of the Ti_3_C_2_T*_x_*/PbS CQDs NIR photodiode is illustrated in **Figure** **1**a. Few‐layered Ti_3_C_2_T*_x_* was synthesized by etching Ti_3_AlC_2_ and subsequent delamination with LiCl (Figure S1 and Experimental Section, Supporting Information). First, a solution‐processed ZnO thin film with controllable roughness of only 0.42 nm was deposited onto the Si/SiO_2_ substrate with Ti/Au bottom electrode to block hole back‐injection (Figures S2 and S3, Supporting Information). Subsequently, PbS CQD was synthesized by a controllable hot injection process.^[^ ^19^ ^]^ As shown in Figure S4 (Supporting Information), the diameter of monodispersed PbS CQD is ≈4 nm and the absorption peak centers at 939 nm, conforming to the quantum limiting effect.^[^ ^20^ ^]^ The photosensitive PbS CQDs layer treated with ligand exchange was directly spin‐coated onto ZnO film. Additionally, the stoichiometric ratio of Pb to S in the PbS CQDs layer treated with 1,2‐ethanedithiol (EDT) was characterized by X‐ray photoelectron spectroscopy (XPS) and determined to be Pb:S = 1.06:1 (Figure S5, Supporting Information). Then, MXene film was sprayed on the top to enable top illumination. It is noteworthy that the spray‐coating process is easier than other coating methods to control the film thickness and continuity, as PbS CQDs surfaces are hydrophobic. The layered architecture was confirmed with the cross‐sectional transmission electron microscope (TEM) images of the as‐prepared Ti_3_C_2_T*_x_*/PbS CQDs photodiode (Figure 1b,e). Furthermore, the energy dispersive spectrometer (EDS) mapping was bestowed to identify the detailed layer components (Figure S6, Supporting Information). The element mapping of Ti and Pb is used to distinguish MXene and PbS layers, indicating that the layer thicknesses are 30 and 230 nm, respectively (Figure 1c; Figure S7, Supporting Information).

Transmittance and electrical conductivity are the key factors of TCE for top‐illuminated photodetectors. For the Ti_3_C_2_T*_x_*/PbS CQD NIR photodiode, the IR light (wavelength range of ≈900−1,500 nm) transmittance of MXene film reaches nearly 80% when the thickness is ≈10 nm. For ≈120 nm‐thick MXene film, the transmittance plungs to less than 20% (Figure S8, Supporting Information). Moreover, the electrical conductivity of as‐deposited Ti_3_C_2_T*_x_* MXene films (≈25−120 nm) is ≈8000 S cm^−1^ (Figure S9, Supporting Information), which is higher than the most traditional TCE materials, such as ITO, carbon nanotubes, and poly(3.4‐ethylenedioxythiophene):poly(styrenensulfonate) (Figure 1d).^[^ ^21^ ^]^ Figure 1e shows the relative contents of the Ti_3_C_2_T*_x_* and PbS CQD layers at different depths. Notably, the contents of Pb or S elements in Ti_3_C_2_T*_x_*/PbS CQDs device increase rapidly to their maximum with a distance of 10 nm, while those in ITO/PbS CQDs reaches the maximum at a distance of 25 nm. It indicates that MXene as the TCE has an ascendant protective effect on the PbS CQDs layer (Figure S10, Supporting Information). The interface engineering with the entire solution processes provides a cornerstone for the subsequent realization of high‐performance Ti_3_C_2_T*_x_*/PbS CQDs NIR photodiodes.

To further verify the reliability, the drive‐level capacitance profiling (DLCP) and capacitance–voltage (*C–V*) measurements were performed to characterize the defects in Ti_3_C_2_T*_x_*/PbS CQDs device.^[^ ^10^ ^]^ The *C–V* profiles show the contribution of both interface and bulk states, while the interface states hardly affect the DLCP curves.^[^ ^22^ ^]^ Therefore, the difference between each pair of curves is associated with the presence of interface defects, and the free carrier concentration can be determined by the DLCP profile. The interfacial defect density of Ti_3_C_2_T*_x_*/PbS CQDs photodiode is estimated as 4.0 × 10^15^ cm^−3^ (Figure 1g; Figure S11, Supporting Information). This value is an order of magnitude lower than that of the ITO/PbS CQDs photodiode with a defect density of 4.0 × 10^16^ cm^−3^ (Figure S12, Supporting Information). Meanwhile, the free carrier concentration of PbS in ITO/PbS CQDs photodiode (1.7 × 10^17^ cm^−3^) exceeds that in Ti_3_C_2_T*_x_*/PbS CQDs photodiode (1.6 × 10^16^ cm^−3^). These suggest that the solution‐processed MXene electrode significantly reduced both the carrier concentration and interface defect density in the PbS photosensitive layer. The low‐defect interface not only results from avoiding the bombardment effects of sputtering but also because MXene can effectively cover the surface cracks present in the CQDs layer.^[^ ^23^ ^]^

**Figure 2:**
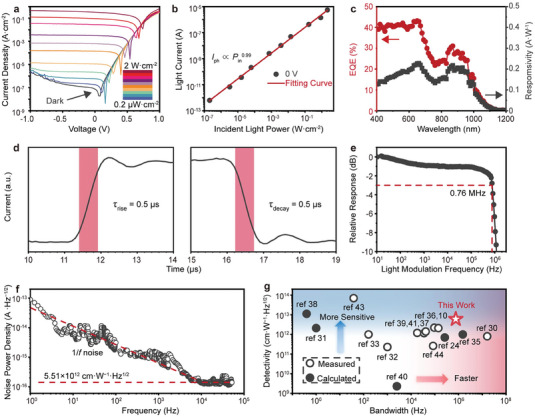
Photoelectric detection performance of Ti_3_C_2_T*_x_*/PbS CQDs NIR photodiode. a) Semilog *J‐V* curves of the device in the dark and under NIR (940 nm) illumination at intensities from 0.2 µW cm*^−^* ^2^ to 2 W cm*^−^* ^2^. b) Linear dependence of generated photocurrents of the Ti_3_C_2_T*_x_*/PbS CQDs NIR photodiode on the utilized 940 nm light intensity. c–e) EQE and responsivity spectra (c), transient response (d), and response bandwidth (e) of the Ti_3_C_2_T*_x_*/PbS CQDs NIR photodiode at zero voltage bias. f) Measured current noise of the Ti_3_C_2_T*_x_*/PbS CQDs NIR photodiode as a function of frequency at zero voltage bias. g) Specific detectivity (D^*^) and bandwidth statistics of the Ti_3_C_2_T*_x_*/PbS CQDs NIR photodiode with typical PbS CQDs photodetectors reported in the literature.

To comprehensively evaluate the performance of Ti_3_C_2_T*_x_*/PbS CQDs photodiodes, the key figures of merit of IR photodetectors were measured. **Figure** **2**a shows the current density–voltage (*J–V*) characteristics of the device under dark conditions with varied 940 nm laser intensities from 0.2 µW cm^−2^ to 2 W cm^−2^. Remarkably, the dark current density is only 0.2 µA cm^−2^ at −0.5 V bias voltage. A lower dark current can improve the sensitivity of the photodetector, enabling the detection of extremely weak light signals. Notably, the device exhibits a high rectification ratio by five orders of magnitude at ±1 V (Figure S13, Supporting Information), indicating low series resistance and efficient charge extraction.^[^ ^24^ ^]^ A reduced rectification ratio of the Ti_3_C_2_T*_x_*/PbS CQDs NIR photodiode was obtained in a vacuum, which is attributed to the changes in the conductivity and *WF* after the 2D MXene adsorbs the foreign species (such as H_2_O, O_2_, etc.) in an ambient environment.^[^ ^25^ ^]^ The photocurrent and open‐circuit voltage of the device increase with higher laser intensity. Specially, the open‐circuit voltage value reaches 0.74 V in the forward scan at a light intensity of 2 W cm^−2^, while the photocurrent remains nearly constant at reverse bias. These demonstrate that the device is suitable for self‐powered operation without an external bias.^[^ ^26^ ^]^ Figure 2b shows the linear relationship between photocurrent and optical power in the self‐powered state (*I*_ph_∝*P*_in_^0.99^). Another important metric for the photodetector is the linear dynamic range (LDR), which represents the photodetector with a constant response to the change in light intensity. It can be calculated by

(1) $$\mathrm{LDR}=20\times \log\left(P_{sat}/P_{low}\right)$$

where *P*_sat_ and *P*_low_ represent the maximum and minimum light intensities at which the photocurrents begin to deviate from linearity. The LDR of the device is 140 dB, which is comparable to the commercial silicon photodetector (160 dB).^[^ ^27^ ^]^ Notably, a large LDR depends on its small noise equivalent power and large saturation light intensity. The saturation is determined by the ratio of carrier lifetime and extracted duration, which finally defines the upper limit of LDR.^[^ ^28^ ^]^ When the carrier concentration injected by light is comparable to the equilibrium majority carrier concentration, the carrier concentration gradient will produce an electric field opposite to the built‐in electric field, reducing the effective electric field and prolonging the extraction time of photogenerated carriers. If the carrier extracted time exceeds the carrier lifetime, the photogenerated carriers cannot be effectively collected, and the photocurrent will be saturated. Due to the low defect states in the bulk and at the interface, the carrier lifetime for Ti_3_C_2_T*_x_*/PbS CQDs photodiode remains long under high light intensity, resulting in a linear response to light intensity up to 2 W cm^−2^.

To quantify the photodetection of the Ti_3_C_2_T*_x_*/PbS CQDs photodiode in the self‐powered state, the responsivity and external quantum efficiency (EQE) at a wavelength from 400 to 1200 nm were measured (Figure 2c). Responsivity quantifies the sensitivity of a photodetector in converting input light signals into electrical signals. Additionally, EQE gauges the efficiency of a photodetector in converting incident light into electrical signals under external illumination, which expresses the ratio of the number of electrons generated per incident photon to the total number of incident photons. Our device exhibits a peak responsivity of 0.19 A W^−1^ and an EQE of 25% at 940 nm. Meanwhile, the statistical results of the responsivity and EQE for MXene/PbS and ITO/PbS CQDs photodiodes under 940 nm illumination at −0.5 V bias voltage are presented in Figure S14 (Supporting Information). Both the responsivity and EQE of the MXene/PbS photodiodes are higher than the ITO/PbS CQDs devices, suggesting that the improved interface with MXene leads to reduced carrier recombination and increased device responsivity.

For a photodetector, a short response time signifies its rapid responsiveness to light signal variations. Traditionally, the response time involves the duration required to recombine the excess carriers and to clear both shallow‐ and deep‐level defects.^[^ ^29^ ^]^ Owing to the elimination of deep defects caused by sputtering, the Ti_3_C_2_T*_x_*/PbS CQDs photodiode exhibits rapid response in the sub‐microsecond range under the self‐powered state. Both the rise and decay time are only 0.5 µs, which were measured between 10% and 90% of the maximum photocurrent (Figure 2d). In addition, the frequency response suggests the fast response of the Ti_3_C_2_T*_x_*/PbS CQDs photodiode with a −3 dB bandwidth of up to 0.76 MHz, illustrating that the device is capable of transmitting high‐frequency signals and responding to rapid changes of the light signals (Figure 2e).

Specific detectivity (D^*^) is the key figure of merit to evaluate the performance of photodetectors, which depends on the responsivity, current noise, and the area of the device. It is defined as

(2) $$D^{*}=\frac{R\sqrt{A}}{i_{n}}$$

where *i*_n_ is the root mean square current noise in a 1 Hz bandwidth, and *A* is the area of the device (2.5 × 10^−5^ cm^2^). To identify the current noise, the frequency‐dependent current noise spectrum of the Ti_3_C_2_T*_x_*/PbS CQDs photodiode at zero bias was measured (Figure 2f). At low frequencies, the noise current decreases with the increasing frequency, which conforms to the characteristic of 1/*f*.^[^ ^10^ ^]^ At higher frequencies (>10 kHz), the noise current stabilizes at ≈1.35 × 10^−16^ A Hz^−1/2^. First, the background noise of the signal analyzer has been measured by disconnecting the devices, which is lower by about one order of magnitude than the detector noise (Figure S15, Supporting Information). According to the noise current and photo response at 10 kHz, the D^*^ value of the Ti_3_C_2_T*_x_*/PbS CQDs photodiode in the self‐powered state is ≈5.51 × 10^12^ cm W^−1^ Hz^1/2^. Moreover, the responsivity and D^*^ under different laser intensities (Figure S16, Supporting Information) show small fluctuations within the linear response range, indicating the stable detection performance of the Ti_3_C_2_T*_x_*/PbS CQDs photodiode in the NIR range. The figures of merits of typical PbS CQDs photodiodes are summarized in Table S1 (Supporting Information) and Figure 2g.^[^ ^10^, ^24^, ^30^, ^31^, ^32^, ^33^, ^34^, ^35^, ^36^, ^37^, ^38^, ^39^, ^40^, ^41^, ^42^, ^43^, ^44^ ^]^ The Ti_3_C_2_T*_x_*/PbS CQDs photodiode showcases higher sensitivity and larger bandwidth, demonstrating the potential in optical communications, on‐chip spectroscopy, and sensing.^[^ ^24^, ^45^ ^]^

**Figure 3:**
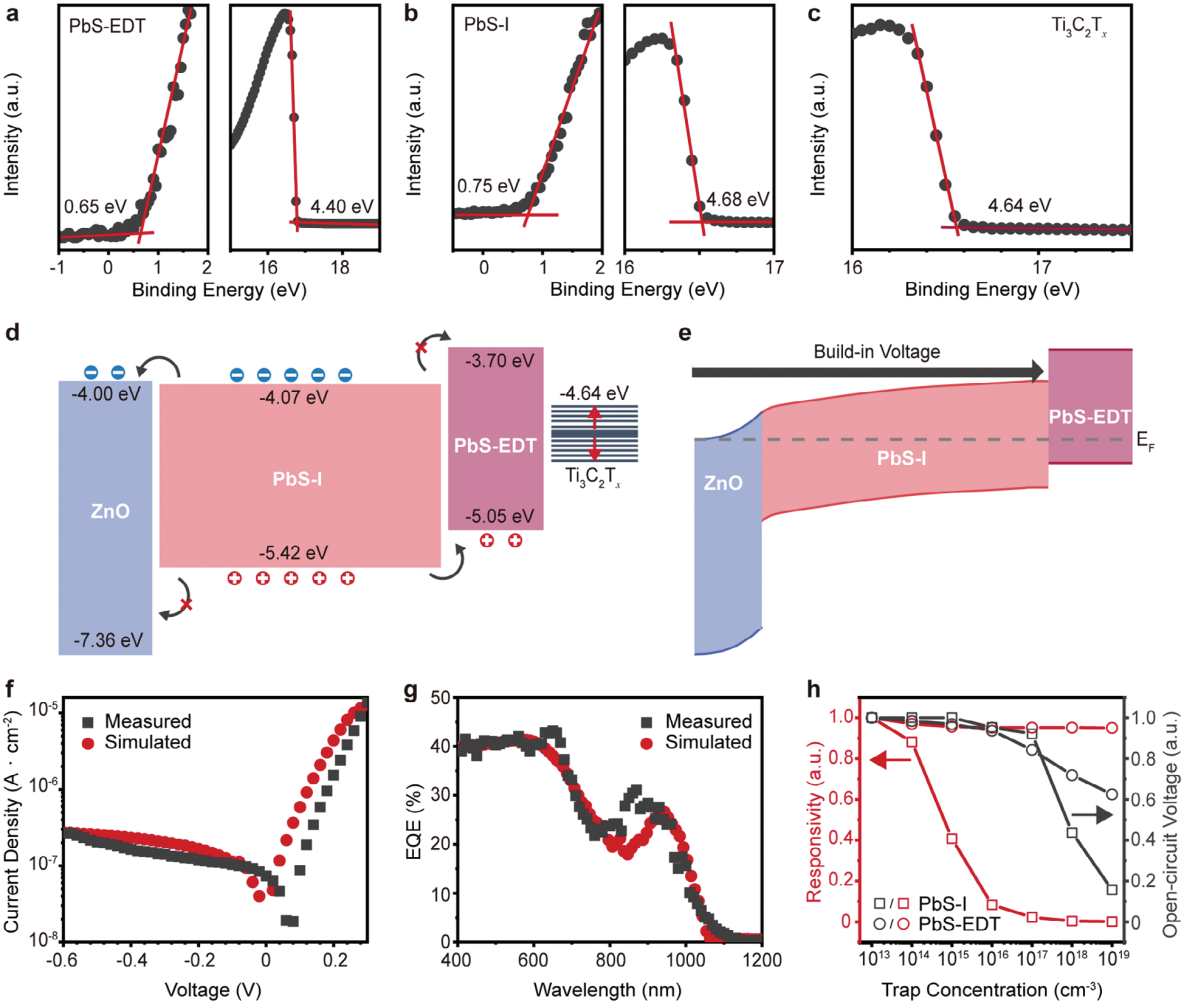
Working mechanism of Ti_3_C_2_T*_x_*/PbS CQDs NIR photodiode. a–c) UPS characterization of a) PbS‐EDT, b) PbS‐I, and c) Ti_3_C_2_T*_x_*, respectively. d) Energy band diagrams of the ZnO, PbS‐I, PbS‐EDT, and Ti_3_C_2_T*_x_* before contact formation. e) Energy level alignment in the thermal‐equilibrium state for Ti_3_C_2_T*_x_*/PbS CQDs NIR photodiode. Comparison of f) dark current density and g) EQE spectra between experimental results and the theoretical model developed using Sentaurus TCAD. h) Normalized responsivity and open‐circuit voltage of PbS‐I and PbS‐EDT layers under 0.1 W cm^−2^ illumination with varying trap concentrations.

To clarify the underlying mechanisms of the Ti_3_C_2_T*_x_*/PbS CQDs photodiode, a comprehensive investigation was conducted to analyze the band alignment in each layer of the device. According to the ultraviolet‐visible absorption spectrum (Figure S17, Supporting Information), the bandgap of ZnO is determined to be 3.36 eV, which is consistent with the previous studies.^[^ ^39^ ^]^ Ultraviolet photoelectron spectroscopy (UPS) was employed to accurately ascertain the Fermi energy levels and valence band maximum (VBM) of ZnO, PbS, and Ti_3_C_2_T*_x_* film deposited on Au/SiO_2_/Si substrates (Figure S18, Supporting Information). Through EDT ligand exchange, the Fermi level of PbS CQDs was measured to be −4.40 eV, with a corresponding VBM of −5.05 eV (**Figure** **3**a). Likewise, the PbS CQDs employed by the iodine ligand exchange process exhibit a Fermi level of −4.68 eV and a VBM of −5.42 eV (Figure 3b). Furthermore, the *WF* of Ti_3_C_2_T*_x_* was determined to be −4.64 eV (Figure 3c), which can be effectively controlled by modifying its surface termination.^[^ ^25^ ^]^ Based on these results, the device band alignment diagram was constructed, as illustrated in Figure 3d. The proximity of the conduction band positions between PbS‐I and ZnO facilitates smooth electron flow from PbS‐I to ZnO. Conversely, compared to PbS‐I, the significantly lower valence band position of ZnO effectively prevents hole reverse flow and electron‐hole recombination. Similarly, PbS‐EDT exhibits higher conduction and valence bands, allowing facile hole transfer from PbS‐I to PbS‐EDT while impeding electron flow into PbS‐EDT.

For Ti_3_C_2_T*_x_*/PbS CQDs photodiode, the energy band diagram at thermal equilibrium reveals the working mechanism of the device in the self‐powered state (Figure 3e). The ZnO/PbS‐I PN junction with different carrier concentrations results in a wide depletion region (Figure S19, Supporting Information). Importantly, the ZnO layer in the photodiode device acts as a hole‐blocking layer and electron‐transporting layer, while the PbS‐EDT layer is the electron‐blocking layer and hole‐transporting layer. It effectively prevents carrier injection from the electrodes into PbS‐I. Consequently, the PbS‐I absorber layer is able to be fully depleted at zero bias. Generally, the response time of a photodiode encompasses fast and slow components, which are the drift time of photo‐generated carriers in the depletion region and quasi‐neutral region, respectively.^[^ ^35^ ^]^ In the absence of a quasi‐neutral region, the built‐in electric field impacts the entire absorber layer, enabling efficient drift and collection of photo‐generated carriers. This unique configuration allows the device to operate in a self‐powered state with rapid photo response. According to the measured energy level of each layer in the device, the dark current density curve and EQE spectra were simulated using the tool Sentaurus Technology computer‐aided design (TCAD), as shown in Figure 3f,g. The detailed parameters used in the simulation are listed in Table S2 (Supporting Information). The overall curve shape for both the dark current density and EQE corresponds well to the measured data, indicating that the theoretical model is reasonable for comparison to the experimental data. Furthermore, as shown in Figure 3h, we simulated the impact of trap concentration on the optical performance within the PbS‐I and PbS‐EDT layers. The open‐circuit voltage of the device decreases with an increasing trap concentration in the PbS‐EDT layer, though the decrease in responsivity is marginal. In contrast, a rise in trap concentration in the PbS‐I layer significantly diminishes the device's responsivity. Therefore, avoiding the introduction of defects in the absorber layer is a pivotal strategy to exploit high‐performance photodiode devices.

**Figure 4:**
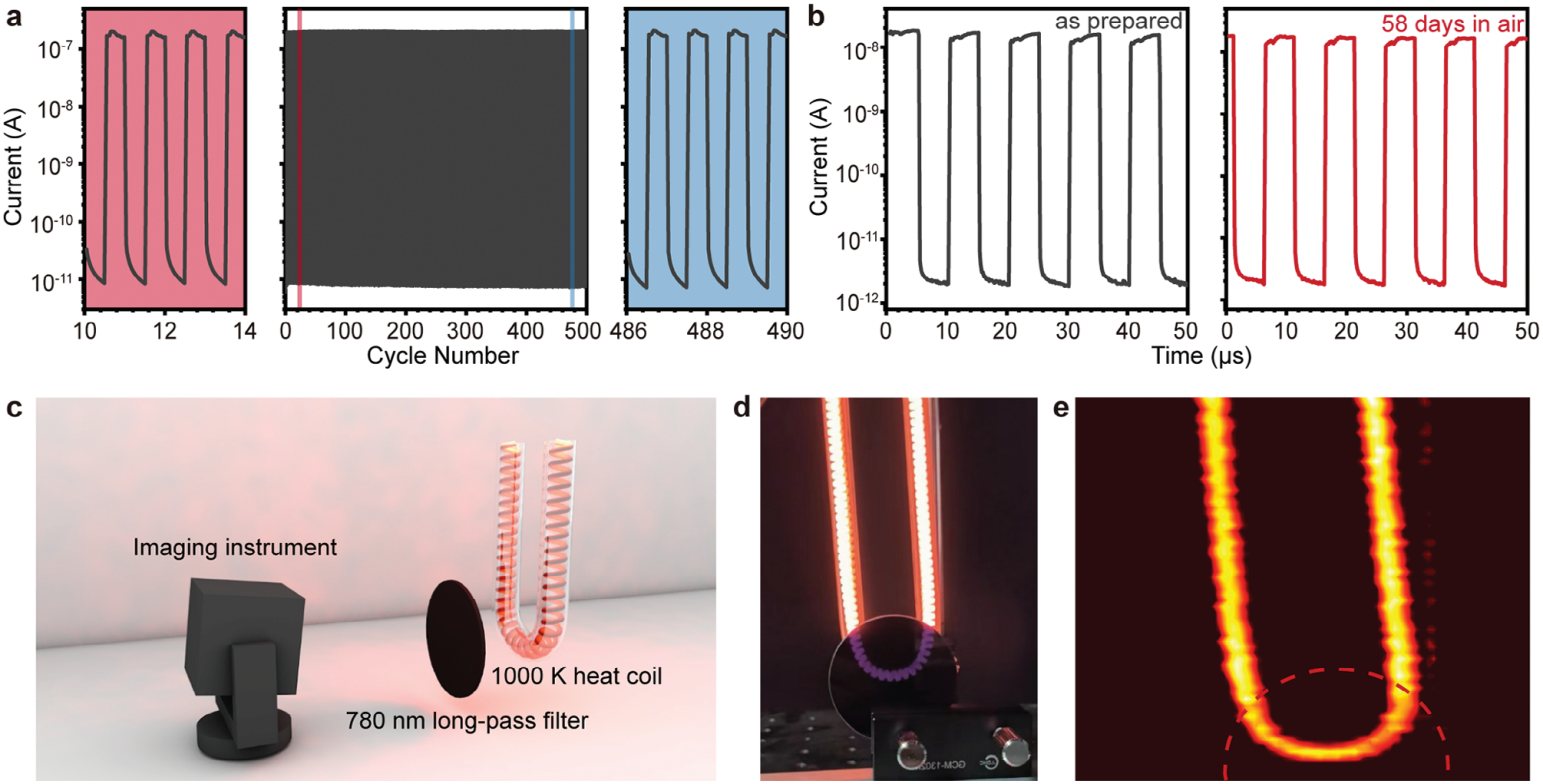
Stability and imaging capability of Ti_3_C_2_T*_x_*/PbS CQDs NIR photodiode. Transient photoresponse of the Ti_3_C_2_T*_x_*/PbS CQDs NIR photodiode a) during operation about 500 cycles and b) before and after storage in ambient conditions for 58 days. c) Schematic diagram of the imaging system for Ti_3_C_2_T*_x_*/PbS CQDs NIR photodiode. d) Photograph of the target (carbon fiber heating tube covered by long‐pass 780 nm filter) was captured by a silica‐based camera. e) NIR image captured using the Ti_3_C_2_T*_x_*/PbS CQDs NIR photodiode in a self‐powered state, with objects behind a long‐pass 780 nm filter marked by a dashed line.

To evaluate the reliability of the photodetector, cyclic testing under 940 nm laser illumination was conducted. The photocurrent and dark current of the Ti_3_C_2_T*_x_*/PbS NIR photodiode remain stable even after 500 cycles (**Figure** **4**a). Furthermore, we exposed the fabricated devices to an ambient atmosphere with 40% humidity at room temperature (25 °C) for 58 days. There is no obvious change in the photocurrent and dark current (Figure 4b), demonstrating the effective protection of the MXene layer on PbS CQDs against oxidation.^[^ ^46^ ^]^

Finally, we investigated the IR imaging capability of the detector. The setup for the IR imaging test, depicted in Figure 4c, includes a 1000 K thermal source (carbon fiber heating coil) with a long‐pass 780 nm filter in the front. The peak radiation wavelength of the thermal source is ≈2 µm according to Planck's blackbody radiation law. An image was acquired by 2D scanning and converting the output photocurrent of each pixel into a gray value. A comparison of the image was captured by a complementary metal‐oxide‐semiconductor transistor chip in a smartphone (Figure 4d). The scanning imaging performance of the MXene/PbS CQDs photodiode in its self‐powered state exhibits similar thermal imaging results for the thermal source, regardless of the presence or absence of the 780 nm filter (Figure 4e). The corresponding photocurrents are presented in Figure S20 (Supporting Information). The peak photocurrent with the filter is only 78% of the peak photocurrent without the filter, indicating a strong photocurrent generated under NIR illumination. Briefly, the MXene/PbS photodiode emerges as a promising candidate for power‐efficient NIR imaging applications.

## 3 Conclusion

In summary, we have successfully fabricated a high‐performance Ti_3_C_2_T*_x_*/PbS CQDs photodiode operating in the NIR region with fully solution‐processed interface engineering. The MXene TCE possesses high electrical conductivity, large work function, and good optical transmittance. The top‐illuminated Au/ZnO/PbS/MXene NIR photodiode device exhibits a dynamic response range of 140 dB, a −3 dB bandwidth of 0.76 MHz, and a detectivity of 5.51 × 10^12^ cm W^−1^ Hz^1/2^ in the self‐powered state under 940 nm illumination at room temperature. Meanwhile, the device with the protection of the MXene layer is stable over 58 days in ambient conditions. High‐performance and stable Ti_3_C_2_T*_x_*/PbS CQDs photodiodes shed light on the potential of MXene and CQDs for fully solution‐processed, flexible, and efficient IR imagers.

## 4 Experimental Section

### Synthesis of Ti_3_C_2_T*_x_* and PbS CQD

Ti_3_C_2_T*_x_* and PbS CQD were prepared by the acid‐etching and hot‐injection synthesis recipe, respectively, as described in Experimental Section (Supporting Information).

### Ligand Exchange

The solution‐phase ligand‐exchange process was carried out in glove box^7^. Lead halides (0.10 m PbI_2_, 0.04 m PbBr_2_) and 0.06 m NH_4_Ac were dissolved in N, N‐Dimethylformamide (DMF) as the ligand solution. PbS CQD (20 mg mL^−1^ in octane) capped with 10 mL oleic acid were added to a ligand solution (10 mL). The biphasic system was vigorously vibrated for 3 min until the complete transfer of PbS CQD from octane to DMF. The PbS CQD DMF solution was then rinsed with hexane three times. The ligand‐exchanged PbS CQD was precipitated and separated by centrifugation with toluene and dried in a vacuum chamber for 30 min. The final CQD were redispersed in mixed solvents (volume ratio of butylamine/DMF as 4/1) with a concentration of 400 mg mL^−1^.

### Device Fabrication

For the fabrication of PbS CQDs photodiodes, a ZnO sol–gel solution was deposited on the pre‐cleaned Si/SiO_2_ substrates by spin‐coating as an electron transport layer and dried at 200 °C for 30 min under the atmosphere. Next, the absorption layer of PbS CQDs was spin‐coated at 1500 rpm for 30 s using ≈400 mg mL^−1^ I^−^/Br^−^‐capped CQDs inks with film thicknesses of 300–500 nm. After the obtained absorption layers were heated at 75 °C for 15 min in the glove box, a 50 nm PbS CQD‐EDT layer was fabricated onto it. The OA‐capped PbS‐CQDs were deposited by spin‐coating at 1500 rpm for 30 s, treated with 0.2% volume ratio of EDT in isopropanol for 30 s, and then washed with isopropanol. Notably, this additional PbS CQD‐EDT layer enabled better absorber film morphology and improved device reproducibility. Finally, the Ti_3_C_2_T*_x_* MXene films were manually spray‐coated as the top conductive electrode. A post‐annealing step at 75 °C for 15 min within a glove box was applied to eliminate H_2_O adsorbed by PbS CQDs. As a reference, the ITO electrodes were deposited by magnetron sputtering at room temperature. The thicknesses of ZnO, PbS‐EDT, PbS‐I, Ti_3_C_2_T*_x_*, and ITO layers were ≈50, 300, 50, and 15 nm, respectively.

### Material Characterization

X‐ray diffraction (XRD) was conducted on a Bruker D8 Discover diffractometer operating with a Cu K α energy source at 40 kV and 40 mA. Bright‐filed TEM images of MXene were taken using Tecnai G2 F20 S‐Twin (FEI) at 300 kV. Atomic force microscope (AFM) images were collected using a commercial Asylum Research system. The optical absorption spectra were collected using a Hitachi U‐4100 Plus spectrophotometer. The sheet resistance of as‐deposited MXene film was measured using an ST‐2258C multifunction digital four‐probe tester. XPS and UPS measurements were performed with the AXIS Ultra DLD in ultrahigh vacuum conditions (10^−10^ mbar) and measured with a monochromatic HeI UV source (21.2 eV). The cross‐section sample of PbS CQDs photodiode devices was treated by a focused ion beam (FIB) lift‐out technique with Tescan GAIA3. The TEM images and EDS results were obtained through a line sweep from the top layer to the down layer by Thermofisher TALOS F200X.

### Device Characterization

The *C–V* and DLCP curves were measured at room temperature using a semiconductor analyzer (Keithley 4980) with the frequency set at 10 kHz. For the *C–V* measurement, the amplitude of the ac bias was 30 mV, and the dc bias was scanned from −1 to 1 V. The DLCP measurement was conducted in the dc bias range of −0.5–0.5 V (or 0.7 V), whereas the amplitude of the ac bias ranged from 10 to 300 mV. The *J–V* curves and time‐resolved photocurrent measurements under dark conditions and the illumination of the representative wavelength of 940 nm were characterized by an MS200 system using a semiconductor analyzer (Keithley 6482). The transient response and −3 dB bandwidth were recorded by an MS200 system using a PicoScope 4262 oscilloscope and a low‐noise current preamplifier (SR570, Stanford Research Systems). The noise spectra of the PbS CQDs photodiode were measured up to 50 kHz using a signal analyzer (Keysight 35670A) combined with a low‐noise current preamplifier powered by electric batteries (SR570, Stanford Research Systems). The EQE values at each wavelength were measured using a semiconductor analyzer (Keithley 6482) and a spectrometer (Horiba iHR350) with a xenon lamp.

## Conflict of Interest

The authors declare no conflict of interest.

## Supporting information

Supporting Information

## Data Availability

Research data are not shared.

## References

[1] S. A. Mcdonald , G. Konstantatos , S. Zhang , P. W. Cyr , E. J. D. Klem , L. Levina , E. H. Sargent , Nat. Mater. 2005, 4, 138. 15640806 10.1038/nmat1299

[2] R. Saran , R. J. Curry , Nat. Photonics 2016, 10, 81.

[3] H. Wang , Y. Dong , X. Fu , X. Zhao , Q. Zhao , M. Xia , M. Kang , C. Zhao , Z. Xu , Y. Zhu , L. Gao , J. Tang , L. Dong , J. Miao , W. Hu , IEEE Trans. Nanotechnol. 2023, 22, 359.

[4] C. G. Granqvist , A. Hultåker , Thin Solid Films 2002, 411, 1.

[5] Z. Wu , Z. Chen , X. Du , J. M. Logan , J. Sippel , M. Nikolou , K. Kamaras , J. R. Reynolds , D. B. Tanner , A. F. Hebard , A. G. Rinzler , Science 2004, 305, 1273. 15333836 10.1126/science.1101243

[6] Y. Song , W. Fang , R. Brenes , J. Kong , Nano Today 2015, 10, 681.

[7] Y. Wang , C. Zhu , R. Pfattner , H. Yan , L. Jin , S. Chen , F. Molina‐Lopez , F. Lissel , J. Liu , N. I. Rabiah , Z. Chen , J. W. Chung , C. Linder , M. F. Toney , B. Murmann , Z. Bao , Sci. Adv. 2017, 3, e1602076. 28345040 10.1126/sciadv.1602076 PMC5345924

[8] S. Ahn , T.‐H. Han , K. Maleski , J. Song , Y.‐H. Kim , M.‐H. Park , H. Zhou , S. Yoo , Y. Gogotsi , T.‐W. Lee , Adv. Mater. 2020, 32, 2000919. 10.1002/adma.202000919 32350958

[9] M. Graetzel , R. A. J. Janssen , D. B. Mitzi , E. H. Sargent , Nature 2012, 488, 304. 22895335 10.1038/nature11476

[10] J. Liu , P. Liu , D. Chen , T. Shi , X. Qu , L. Chen , T. Wu , J. Ke , K. Xiong , M. Li , H. Song , W. Wei , J. Cao , J. Zhang , L. Gao , J. Tang , Nat. Electron. 2022, 5, 443.

[11] M. Naguib , M. Kurtoglu , V. Presser , J. Lu , J. Niu , M. Heon , L. Hultman , Y. Gogotsi , M. W. Barsoum , Adv. Mater. 2011, 23, 4248. 21861270 10.1002/adma.201102306

[12] M. Naguib , O. Mashtalir , J. Carle , V. Presser , J. Lu , L. Hultman , Y. Gogotsi , M. W. Barsoum , ACS Nano 2012, 6, 1322. 22279971 10.1021/nn204153h

[13] M. Han , D. Zhang , C. E. Shuck , B. Mcbride , T. Zhang , R. Wang , K. Shevchuk , Y. Gogotsi , Nat. Nanotechnol. 2023, 18, 373. 36646826 10.1038/s41565-022-01308-9

[14] A. D. Dillon , M. J. Ghidiu , A. L. Krick , J. Griggs , S. J. May , Y. Gogotsi , M. W. Barsoum , A. T. Fafarman , Adv. Funct. Mater. 2016, 26, 4162.

[15] M. Han , D. Zhang , A. Singh , T. Hryhorchuk , C. Eugene Shuck , T. Zhang , L. Bi , B. Mcbride , V. B. Shenoy , Y. Gogotsi , Mater. Today 2023, 64, 31.

[16] K. Montazeri , M. Currie , L. Verger , P. Dianat , M. W. Barsoum , B. Nabet , Adv. Mater. 2019, 31, 1903271. 10.1002/adma.201903271 31523860

[17] J. Zhang , N. Kong , S. Uzun , A. Levitt , S. Seyedin , P. A. Lynch , S. Qin , M. Han , W. Yang , J. Liu , X. Wang , Y. Gogotsi , J. M. Razal , Adv. Mater. 2020, 32, 2001093. 10.1002/adma.202001093 32309891

[18] L. Luo , Y. Huang , K. Cheng , A. Alhassan , M. Alqahtani , L. Tang , Z. Wang , J. Wu , Light: Sci. Appl. 2021, 10, 177. 34471092 10.1038/s41377-021-00619-1 PMC8410839

[19] M. Albaladejo‐Siguan , D. Becker‐Koch , E. C. Baird , Y. J. Hofstetter , B. P. Carwithen , A. Kirch , S. Reineke , A. A. Bakulin , F. Paulus , Y. Vaynzof , Adv. Energy Mater. 2022, 12, 2202994.

[20] K. Ba , J. Wang , Mater. Today 2022, 58, 119.

[21] T. Guo , D.i Zhou , S. Deng , M. Jafarpour , J. Avaro , A. Neels , J. Heier , C. Zhang , ACS Nano 2023, 17, 3737. 36749603 10.1021/acsnano.2c11180 PMC9979651

[22] J. T. Heath , J. D. Cohen , W. N. Shafarman , J. Appl. Phys. 2004, 95, 1000.

[23] H. R. You , S. Lee , D. H. Lee , G. Murali , A. S. Nissimagoudar , Y. Kim , S. Park , J. Lee , S. J. Kim , J. Y. Park , B. J. Moon , Y. H. Park , S.‐K. Kim , H. N. Yu , H. J. Kim , W. Lee , G. Ham , H. Lee , S.‐C. Lee , H. Cha , J. Lim , Y. Gogotsi , T. K. An , I. In , J. Choi , Adv. Energy Mater. 2023, 13, 2301648.

[24] J. M. Pina , M. Vafaie , D. H. Parmar , O. Atan , P. Xia , Y. Zhang , A. M. Najarian , F. P. G. De Arquer , S. Hoogland , E. H. Sargent , Nano Lett. 2022, 22, 6802. 35969869 10.1021/acs.nanolett.2c02756

[25] T. Schultz , N. C. Frey , K. Hantanasirisakul , S. Park , S. J. May , V. B. Shenoy , Y. Gogotsi , N. Koch , Chem. Mater. 2019, 31, 6590.

[26] Z. Liu , J. K. El‐Demellawi , O. M. Bakr , B. S. Ooi , H. N. Alshareef , ACS Nano 2022, 16, 7904. 35491863 10.1021/acsnano.2c00558

[27] C. Fuentes‐Hernandez , W.‐F. Chou , T. M. Khan , L. Diniz , J. Lukens , F. A. Larrain , V. A. Rodriguez‐Toro , B. Kippelen , Science 2020, 370, 698. 33154137 10.1126/science.aba2624

[28] C. Bao , Z. Chen , Y. Fang , H. Wei , Y. Deng , X. Xiao , L. Li , J. Huang , Adv. Mater. 2017, 29, 1703209. 10.1002/adma.201703209 28846818

[29] J. Jiang , C. Ling , T. Xu , W. Wang , X. Niu , A. Zafar , Z. Yan , X. Wang , Y. You , L. Sun , J. Lu , J. Wang , Z. Ni , Adv. Mater. 2018, 30, 1804332. 10.1002/adma.201804332 30168633

[30] M. Vafaie , J. Z. Fan , A. M. Najarian , O. Ouellette , L. K. Sagar , K. Bertens , B. Sun , F. P. García De Arquer , E. H. Sargent , Matter 2021, 4, 1042.

[31] J. Yoo , S. Jeong , S. Kim , J. H. Je , Adv. Mater. 2015, 27, 1712. 25613836 10.1002/adma.201404945

[32] R. Dong , C. Bi , Q. Dong , F. Guo , Y. Yuan , Y. Fang , Z. Xiao , J. Huang , Adv. Opt. Mater. 2014, 2, 549.

[33] S. Goossens , G. Navickaite , C. Monasterio , S. Gupta , J. J. Piqueras , R. Pérez , G. Burwell , I. Nikitskiy , T. Lasanta , T. Galán , E. Puma , A. Centeno , A. Pesquera , A. Zurutuza , G. Konstantatos , F. Koppens , Nat. Photonics 2017, 11, 366.

[34] G. Konstantatos , Nat. Commun. 2018, 9, 5266. 30531824 10.1038/s41467-018-07643-7 PMC6288162

[35] J. P. Clifford , G. Konstantatos , K. W. Johnston , S. Hoogland , L. Levina , E. H. Sargent , Nat. Nanotechnol. 2009, 4, 40. 19119281 10.1038/nnano.2008.313

[36] S. Lu , P. Liu , J. Yang , S. Liu , Y. Yang , L. Chen , J. Liu , Y. Liu , B. Wang , X. Lan , J. Zhang , L. Gao , J. Tang , ACS Appl. Mater. Interfaces 2023, 15, 12061. 36848237 10.1021/acsami.2c22774

[37] B. N. Pal , I. Robel , A. Mohite , R. Laocharoensuk , D. J. Werder , V. I. Klimov , Adv. Funct. Mater. 2012, 22, 1741.

[38] Y. Wei , Z. Ren , A. Zhang , P. Mao , H. Li , X. Zhong , W. Li , S. Yang , J. Wang , Adv. Funct. Mater. 2018, 28, 1706690.

[39] J. R. Manders , T.‐H. Lai , Y. An , W. Xu , J. Lee , D. Y. Kim , G. Bosman , F. So , Adv. Funct. Mater. 2014, 24, 7205.

[40] T. Rauch , M. Böberl , S. F. Tedde , J. Fürst , M. V. Kovalenko , G. Hesser , U. Lemmer , W. Heiss , O. Hayden , Nat. Photonics 2009, 3, 332.

[41] R. Sliz , M. Lejay , J. Z. Fan , M.‐J. Choi , S. Kinge , S. Hoogland , T. Fabritius , F. P. García De Arquer , E. H. Sargent , ACS Nano 2019, 13, 11988. 31545597 10.1021/acsnano.9b06125

[42] V. Pejovic , J. Lee , E. Georgitzikis , Y. Li , J. H. Kim , I. Lieberman , P. E. Malinowski , P. Heremans , D. Cheyns , IEEE Electron Device Lett. 2021, 42, 1196.

[43] J. W. Lee , D. Y. Kim , F. So , Adv. Funct. Mater. 2015, 25, 1233.

[44] Q. Xu , I. T. Cheong , H. Song , V. Van , J. G. C. Veinot , X. Wang , ACS Photonics 2022, 9, 2792.

[45] H. Jiao , X. Wang , S. Wu , Y. Chen , J. Chu , J. Wang , Appl. Phys. Rev. 2023, 10, 011310.

[46] A. R. Kirmani , A. D. Sheikh , M. R. Niazi , M. A. Haque , M. Liu , F. P. G. De Arquer , J. Xu , B. Sun , O. Voznyy , N. Gasparini , D. Baran , T. Wu , E. H. Sargent , A. Amassian , Adv. Mater. 2018, 30, 1801661. 10.1002/adma.201801661 29978514

