# Supplementary material for: Interface Engineering to Drive High‐Performance MXene/PbS Quantum Dot NIR Photodiode
Source: Adv Sci (Weinh). 2023 Dec 3;11(6):2307169. doi: 10.1002/advs.202307169 (PMC10853715; doi:10.1002/advs.202307169)
Supplement: Supplementary file 1 — Supporting Information [file ADVS-11-2307169-s001.pdf]

ADVANCED SCIENCE
Open Access

Supporting Information

for *Adv. Sci.*, DOI 10.1002/advs.202307169

Interface Engineering to Drive High-Performance MXene/PbS Quantum Dot NIR Photodiode

Yunxiang Di, Kun Ba, Yan Chen, Xudong Wang, Mingqing Zhang, Xinning Huang, Yi Long, Mengdi Liu, Shukui Zhang, Weiyi Tang, Zhangcheng Huang, Tie Lin, Hong Shen, Xiangjian Meng, Meikang Han*, Qi Liu and Jianlu Wang**

Supporting Information

Interface Engineering to Drive High-Performance MXene/PbS Quantum Dot NIR Photodiode

Yunxiang Di[1,#], Kun Ba[1,#,*], Yan Chen[2], Xudong Wang[3], Mingqing Zhang[2,6], Xinning Huang[3], Yi Long[1], Mengdi Liu[6], Shukui Zhang[5], Weiyi Tang[1], Zhangcheng Huang[1], Tie Lin[3], Hong Shen[3], Xiangjian Meng[3], Meikang Han[2,*], Qi Liu[1,4], Jianlu Wang[1,2,3,4,5,*]

[1]State Key Laboratory of Integrated Chips and Systems, Frontier Institute of Chip and System, Fudan University, Shanghai 200433, China

[2]Institute of Optoelectronics, Shanghai Frontier Base of Intelligent Optoelectronics and Perception, Fudan University, Shanghai 200433, China

[3]State Key Laboratory of Infrared Physics, Shanghai Institute of Technical Physics, Chinese Academy of Sciences, Shanghai 200083, China

[4]Shanghai Qi Zhi Institute, 41st Floor, AI Tower, No. 701 Yunjin Road, Xuhui District, Shanghai 200232, China

[5]Hangzhou Institute for Advanced Study University, Chinese Academy of Sciences, Hangzhou, Zhejiang 310024, China

[6]Department of Materials Science, Fudan University, Shanghai 200433, China

[#]These authors contributed equally: Yunxiang Di, Kun Ba

[*]Corresponding:

kun_ba@fudan.edu.cn; mkhan@fudan.edu.cn; jianluwang@fudan.edu.cn

Methods

Synthesis of $Ti_3C_2T_x$. 1 g Ti_3AlC_2 powder was added into the mixed etching solution of concentrated HCl (12 mL), HF (2 mL), and deionized water (6 mL) at 35 °C for 24

h with stirring. The reacted suspension was washed with deionized water through several rounds of centrifugation and decantation until a pH value of 6−7 was reached. 20 mg mL^{-1} LiCl solution was added into the above $Ti_3C_2T_x$ suspension and subsequently kept oscillating for 30 min. Then, the suspension was washed by centrifugation until a pH of 6−7 was reached. After the clay was swelled, the suspension was collected after centrifugation at 7,500 rpm for 3 min and used for the thin film preparation.

Synthesis of PbS CQDs. PbS CQDs were prepared by the hot-injection synthesis recipe, as described in the previous work[1]. PbO (0.45 g), oleic acid (OA, 1.5 mL), and octadecene (ODE, 18 mL) were degassed in a 10^{-3} mbar vacuum under 100 °C using Schlenk techniques. After 2 h, bis(trimethylsilyl) sulfide (TMS_2S, 180 µL in 5 mL ODE) was swiftly injected into the clear solution under N_2 atmosphere. Instantly the nucleation started, and the color changed to be dark brown. The resulted PbS CQDs were purified using hexane and ethanol and redispersed in octane at a concentration of 50 mg mL^{-1}.

Device characterization

Linear dynamic range (LDR) is calculated by

$$\mathrm{LDR} = 20 \times \lg(P_{sat}/P_{low}) \quad (1)$$

where P_{sat} and P_{low} represent the maximum and minimum light intensities at which the photocurrents begin to deviate from linearity.

Responsivity (R) is commonly expressed in amperes per watt (A W^{-1}), and is calculated by

$$\mathrm{R} = I_{ph}/P_{in} \quad (2)$$

where I_{ph} refers to the net photocurrent obtained by excluding the dark current, P_{in} is incident optical power.

External quantum efficiency (EQE) is calculated by

$$\mathrm{EQE} = R \times hc/\lambda \quad (3)$$

where λ is the light wavelength, h is the Planck constant, and c is the velocity of light. Specific detectivity (D^*) is defined as

$$D^* = \frac{R\sqrt{A}}{i_n} \quad (4)$$

where i_{n} is the root mean square current noise in a 1 Hz bandwidth, and A is the area of the device (2.5×10^{-5} cm^2). The calculation of the device's detectivity at 0 V and 10 kHz is as follows:

$$D^* = \frac{0.19\,\frac{A}{W}\times 0.78\times\sqrt{2.5\times10^{-5}\ cm^2}}{1.35\times10^{-16}\ \mathrm{A}/\sqrt{HZ}} = 5.51\times10^{12}\ \mathrm{cm\cdot W^{-1}\cdot Hz^{1/2}}$$

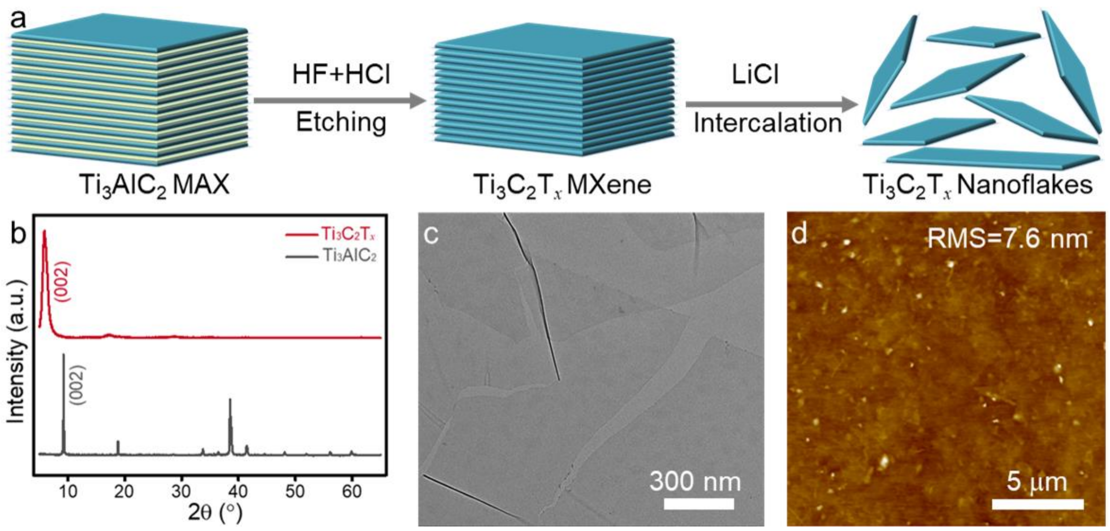

Figure S1. The characterization of as-synthesized $Ti_3C_2T_x$. (**a**) Schematic diagram of the synthesis of few-layered $Ti_3C_2T_x$. (**b**) XRD characterization of $Ti_3C_2T_x$ films. (**c**) TEM image of the single-layer $Ti_3C_2T_x$ flakes. (**d**) Roughness characterization (~7.6 nm) of spray-coated $Ti_3C_2T_x$ films.

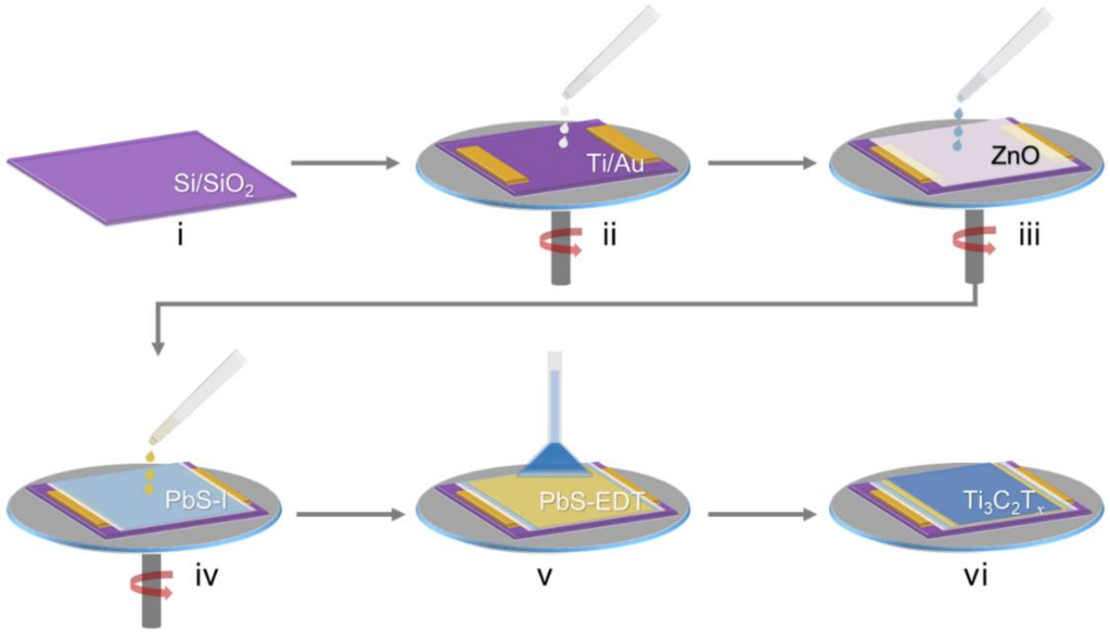

Figure S2. Schematic diagram of the fabrication process for MXene/PbS CQD NIR photodiode.

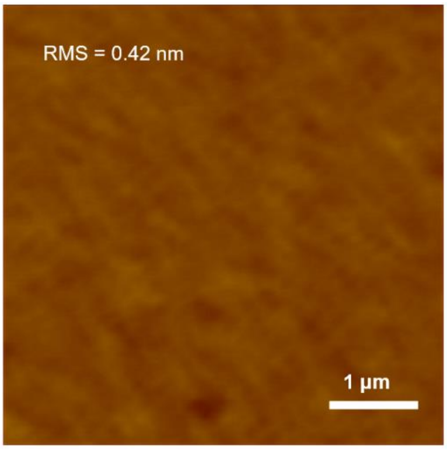

Figure S3. AFM topography and roughness analysis of ZnO film through spin-coating process.

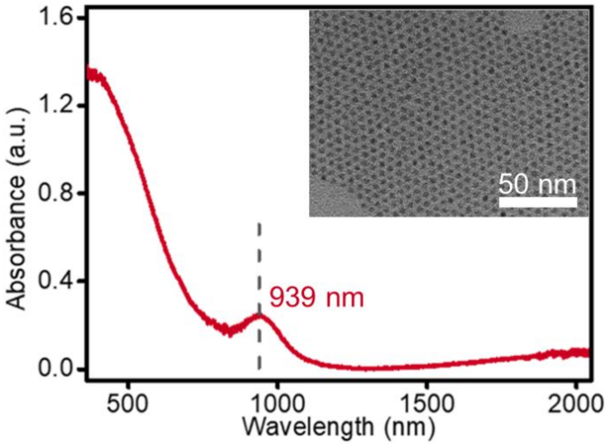

Figure S4. UV-Vis absorption spectroscopy of PbS CQD film. Insert: the TEM image of the as-synthesized OA capped PbS CQDs.

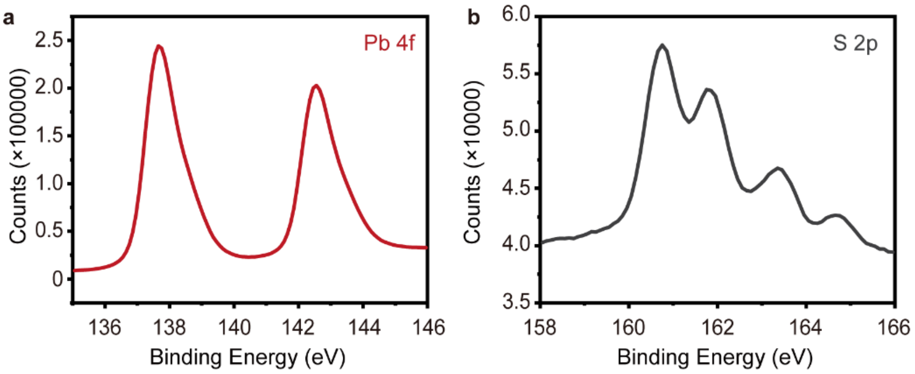

Figure S5. XPS measurements of the Pb (a) and S (b) elements after EDT ligand exchange.

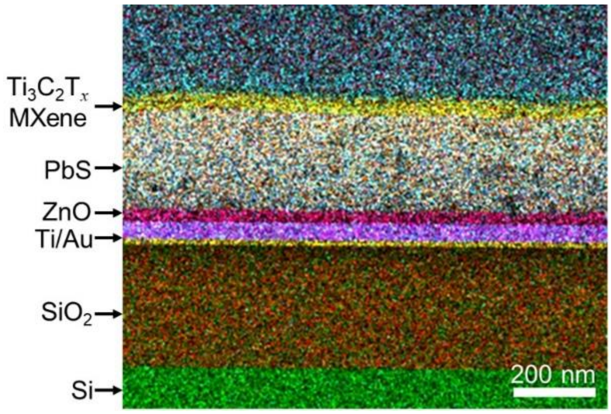

Figure S6. EDS elemental mapping of Si (green), Au (violet), Zn (pink), Pb (brown) and Ti (yellow) of the $Ti_3C_2T_x$/PbS CQDs NIR photodiode.

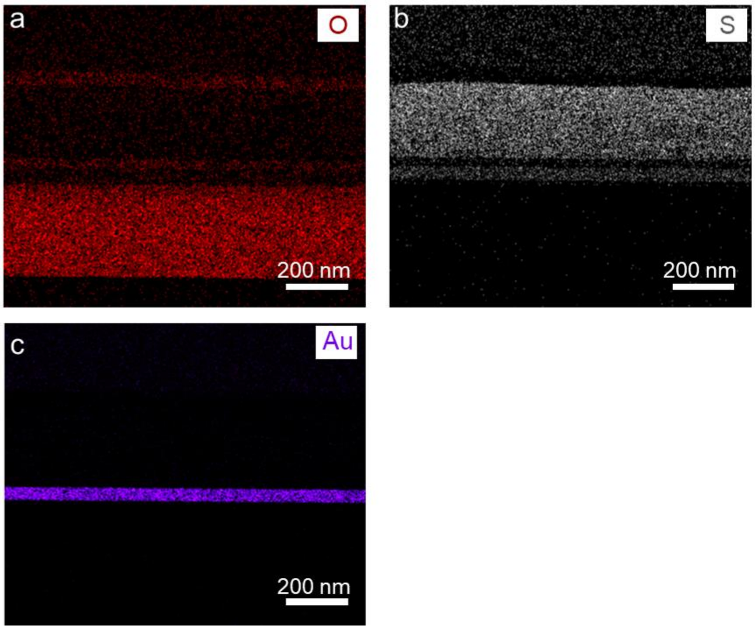

Figure S7. EDS elemental mapping of (**a**) oxygen (red), (**b**) sulfur (white), and (**c**) gold (violet) of the $Ti_3C_2T_x$/PbS CQDs NIR photodiode.

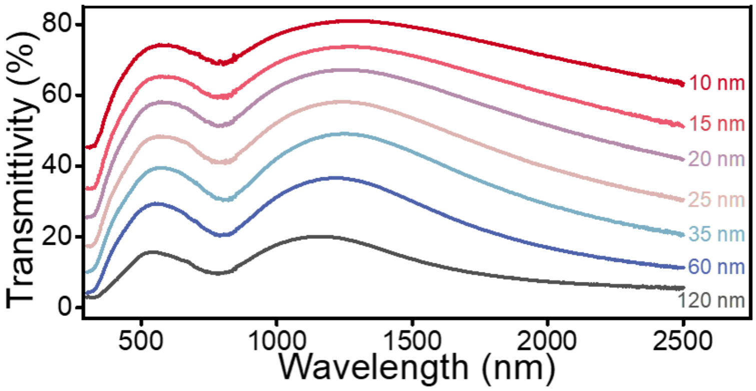

Figure S8. Transmittance characterization of the spray-coated $Ti_3C_2T_x$ films with different thicknesses.

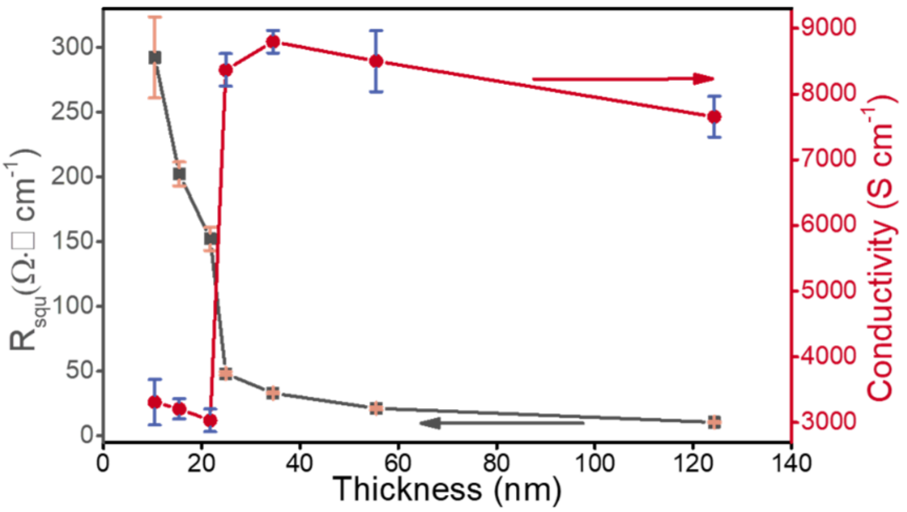

Figure S9. Thickness-dependent sheet resistance and conductivity of the spray-coated $Ti_3C_2T_x$ films.

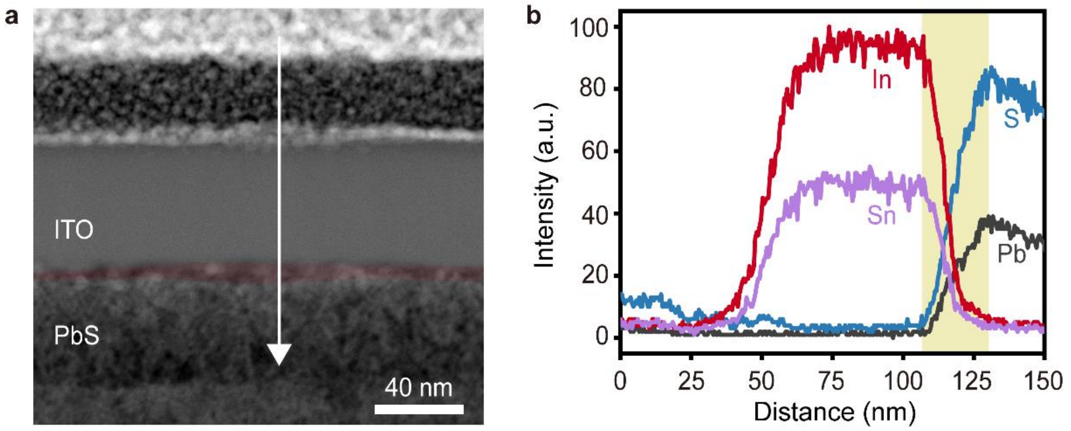

Figure S10. (**a**) Cross-section TEM image and (**b**) element distribution profiles of the ITO/PbS CQD interface. The damaged layer of approximately 5 to 10 nm at the interface between ITO and PbS CQDs is marked in red.

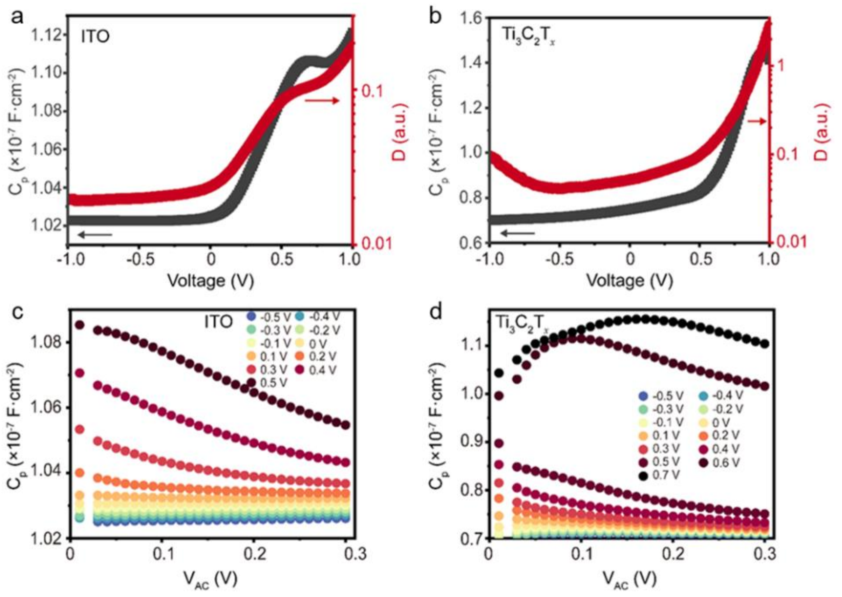

Figure S11. Capacitance and dissipation factor characteristic curves of ITO/PbS CQD (**a**) and $Ti_3C_2T_x$/PbS CQD (**b**) NIR photodiodes by *C-V* measurement. AC bias dependent capacitance of ITO/PbS CQD (**c**) and MXene/PbS CQD (**d**) NIR photodiodes by DLCP measurement. The maximum applied bias is ranging from −0.5 to 0.5 V (or 0.7 V) with a step of 0.1 V.

The defects density obtained from capacitance-voltage (*C-V*) measurement reveals both interface and bulk traps[2]. It is calculated by

$$N_{CV} = -\frac{C^3}{q\varepsilon_0\varepsilon_r A^2\left(\frac{dC}{dV}\right)} = -\frac{2}{q\varepsilon_0\varepsilon_r A^2}\left[\frac{d\left(1/C^2\right)}{dV}\right]^{-1} \quad (5)$$

where q is the elementary charge, ε_r is the relative dielectric constant of PbS CQD (whose value is 23), ε_0 is the vacuum permittivity, A is active area of PbS CQD photodiodes, C is the barrier capacitance. The distance from the heterojunction is calculated as

$$x_d = \varepsilon_0\varepsilon_r A/C \quad (6)$$

The drive-level capacitance profiling (DLCP) technique provides an accurate assessment of the free carrier density in the film. DLCP measurement requires the peak voltage to remain constant. Namely, as the amplitude of the ac voltage (V_{ac}) is adjusted over the course of the experiment, the dc bias (V_{dc}) must simultaneously be adjusted so that the maximum applied voltage V_{ac} + V_{dc} stays constant. In DLCP, the charge response is assumed to be quadratic with V_{ac}:

$$\frac{dQ}{dV} = C_0 + C_1 dV + C_2(dV)^2 \quad (7)$$

where C_0, C_1 and C_2 are the Fourier series of charge response. The defects density is calculated by

$$N_{DL} = -\frac{C_0^3}{2q\varepsilon_0\varepsilon_r A^2 C_1} \quad (8)$$

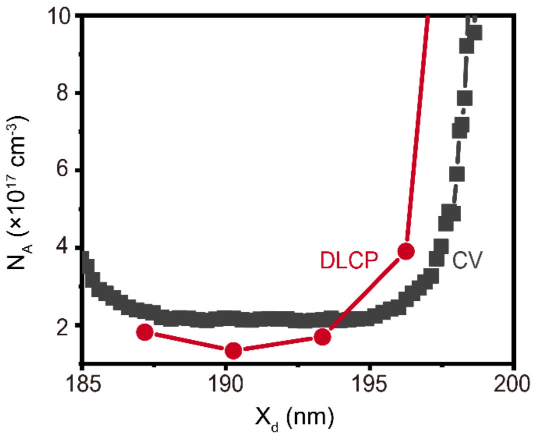

Figure S12. Defect density profiles of ITO/PbS CQDs NIR photodiode by *C*-*V* and DLCP measurement.

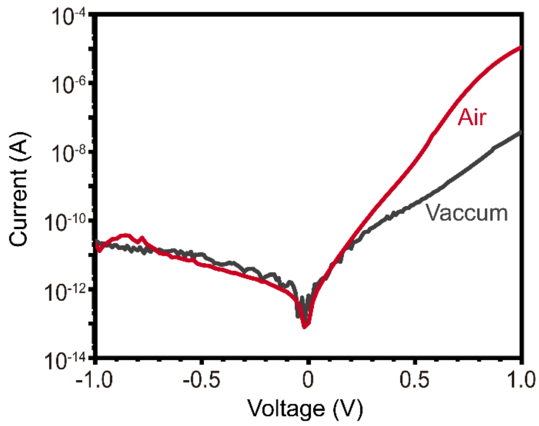

Figure S13. Dark current-voltage (*I*-*V*) characteristics of the device under vacuum and ambient condition.

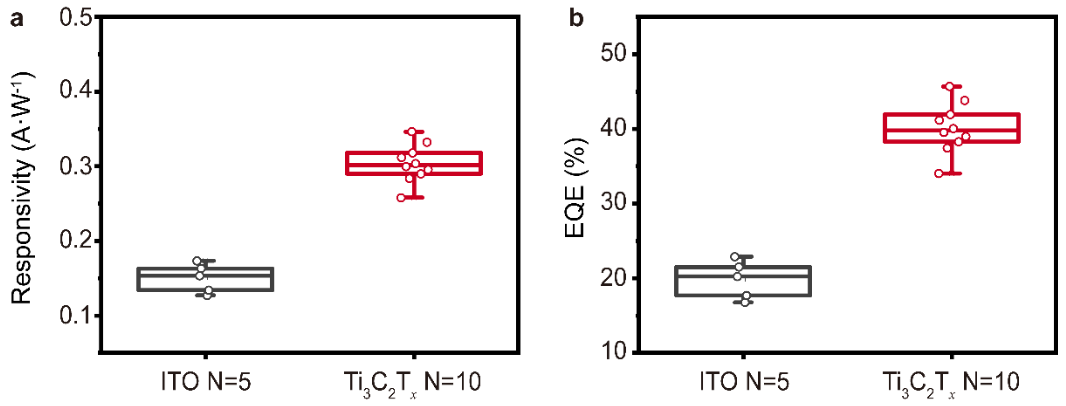

Figure S14. Responsivity (**a**) and EQE (**b**) statistics for ITO/PbS and $Ti_3C_2T_x$/PbS CQD NIR photodiodes at −0.5 V bias voltage under 940 nm illumination.

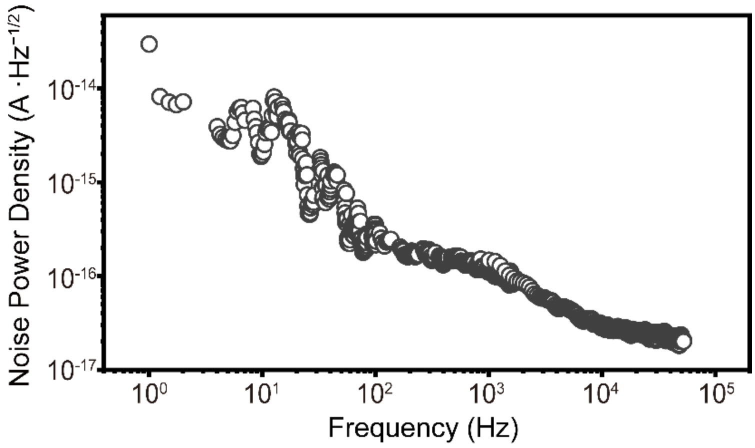

Figure S15. The instrument background noise spectra measured without $Ti_3C_2T_x$/PbS CQD NIR photodiodes.

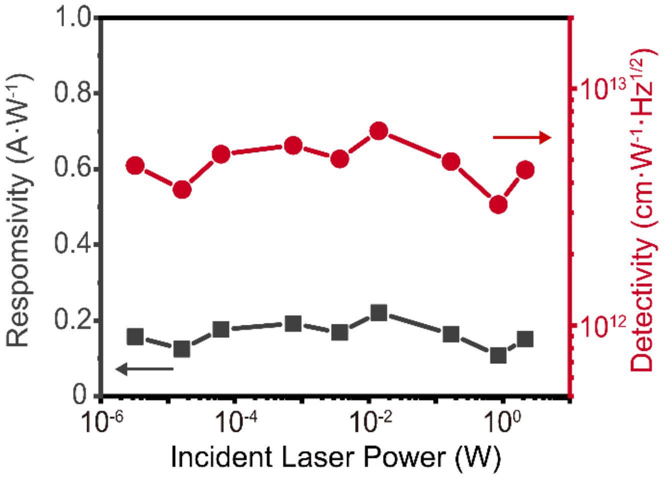

Figure S16. Responsivity and detectivity of the $Ti_3C_2T_x$/PbS CQD NIR photodiode under different laser intensities.

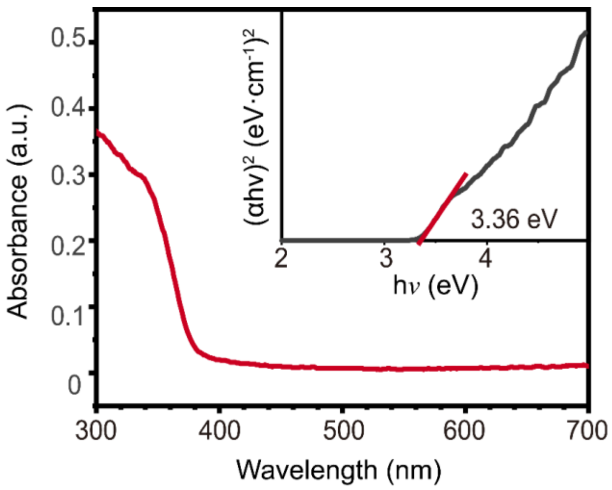

Figure S17. UV-Vis absorption spectrum of ZnO film. Insert: the calculation of the corresponding band gap of ZnO film, whose value is about 3.36 eV.

The optical band gap can be calculated by the conventional Tauc equation[3]:

$$ahv = A(hv - E_g)^n \quad (9)$$

where α is the absorption coefficient, hv is the photon energy, A is a proportionality constant, and n=1/2 for a direct bandgap. The direct bandgap value E_g can be extrapolated by fitting a straight line to the linear segment to intersect the hv-axis from the plot of $(\alpha hv)^2$ against hv, as shown in the insert plot.

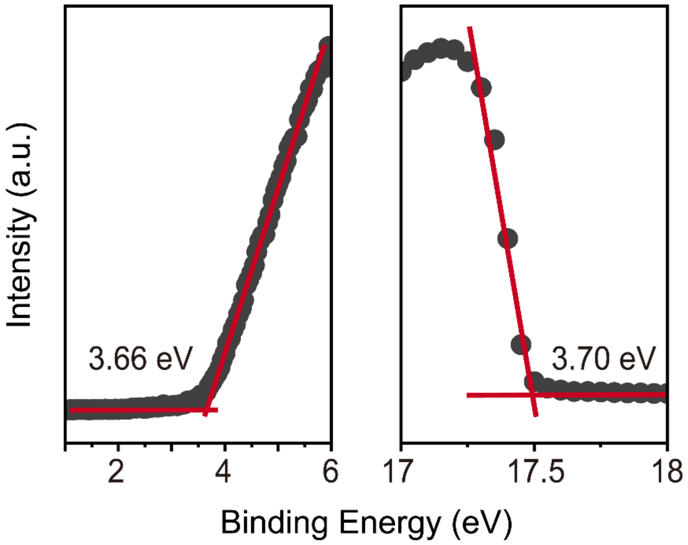

Figure S18. UPS analysis of the ZnO layer deposited by using spin-coating process on Au/SiO_2/Si substrate.

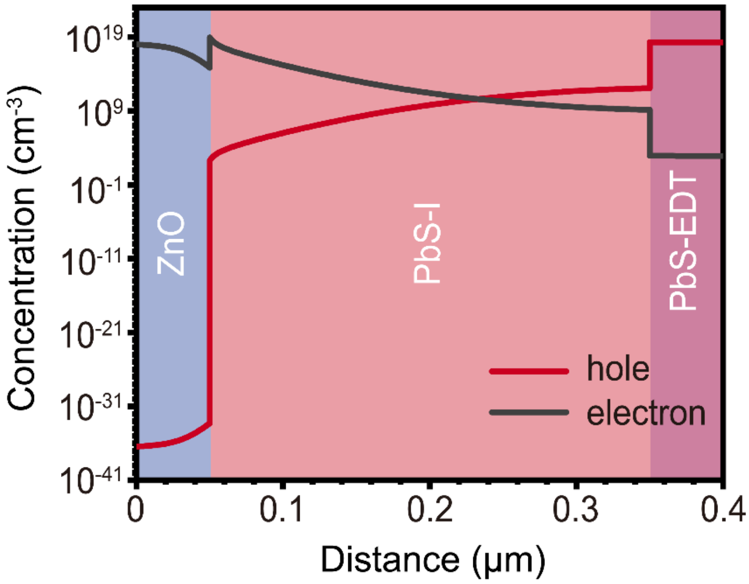

Figure S19. Carrier concentration distribution plot of the device obtained using the tool Sentaurus TCAD.

For an N^+P junction in thermal equilibrium, the depletion layer width can be calculated using the following equation:

$$W = \sqrt{\frac{2\varepsilon_r\varepsilon_0V_{bi}}{eN_A}} \quad (10)$$

where W is the width of depletion layer, q is the elementary charge, ε_r is the relative dielectric constant of PbS CQD (whose value is 23), ε_0 is the vacuum permittivity, V_{bi} bulid-in voltage (around 0.77 V), and N_A is the accepter concentration in PbS CQD layer (around 1×10^{16} cm^{-3}). The estimated depletion layer width is approximately 440 nm, exceeding the width of the absorption layer (300 nm).

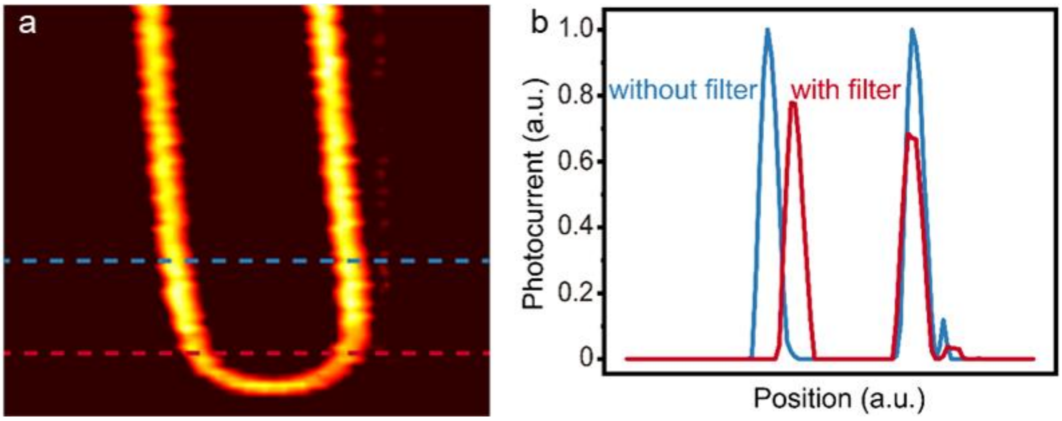

Figure S20. (**a**) Imaging of a heat coil by the $Ti_3C_2T_x$/PbS CQD NIR photodiode at 0 V bias. (**b**) Normalized photocurrent profiles along the horizontal direction of the heat coil.

Table S1. Summary of recently reports of PbS CQD photodetectors.

Device structure	Spectral range (nm)	Detectivity ($cm \cdot W^{-1} \cdot Hz^{1/2}$)	Operation voltage (V)	Bandwidth (Hz)	LDR (dB)	Dark Current Density @-0.5 V (A/cm^2)	Light direction	Year/Ref.
**ITO/PbS/Al**	400−1800	1×10^{12} [C]	0	1.5 M	>60	1×10^{-6}	Back	2009/[4]
**Al/PbS/Au**	300−1200	2.1×10^{12} [C]	−10	1	/	/	Back	2015/[5]
**ITO/PEDOT/PbS-ZnO/Al**	300−1100	2.3×10^{11} [M]	−3	1 K	70	1×10^{-6}	Back	2014/[6]
**ITO/PEDOT/PbS/ZnO/Al**	300−1100	1.2×10^{12} [M]	−1.5	35 K	60	2×10^{-8}	Back	2012/[7]
**ITO/ZnO/PbS/TAPC/MoO_3/Ag**	800−1600	7×10^{13} [M]	−10	38	/	1×10^{-8}	Back	2015/[8]
**ITO/NiO/PbS/ZnO/Al**	400−1400	1.2×10^{12} [M]	−1	18 K	67	2×10^{-8}	Back	2014/[9]
**Glass/PbS/organic/Al**	400−1000	1.1×10^{13} [C]	40	0.4	65	/	Back	2018/[10]
**ITO/ZnO/PbS/spiro/Au**	400−1200	1.4×10^{12} [M]	−1	41 K	>60	2×10^{-7}	Back	2019/[11]
**ITO/ZnO/PbS/Au**	350−1700	8×10^{11} [M]	0	16 M	/	2×10^{-6}	Back	2021/[12]
**ITO/PbS/Ag**	800−1500	7×10^{11} [M]	0	260 K	120	4×10^{-7}	Back	2022/[13]

**ITO/ZnO/PbS/Au**	400−1500	2.2×10^{12} [M]	0	94.6 K	84	1×10^{-7}	Back	2023/[14]
**PEDOT/PbS-organic/Al**	~1500	2.3×10^{9} [C]	−5	2.5 K	40	1×10^{-8}	Top	2009/[15]
**Graphene/PbS**	2000	1×10^{12} [M]	/	160	>80	/	Top	2017/[16]
**NiO/PbS/C_{60}/ZnO/ITO**	400−1300	2.1×10^{12} [M]	−0.5	140 K	>100	1×10^{-8}	Top	2022/[17]
**Si/PbS/ZnO/ITO**	800−1500	2.6×10^{11} [C]	−1	84 K	46	/	Top	2022/[18]
**ZnO/PbS/MXene**	400−1100	5.51×10^{12} [M]	0	0.76 M	140	2×10^{-7}	Top	This work

Note: [C] represents the calculated detectivity using dark current, [M] represents the measured detectivity using noise current.

Table S2. The basic parameters of ZnO, PbS-I, and PbS-EDT layer for simulation.

Parameter	ZnO	PbS-I	PbS-EDT
Thickness (nm)	50	300	50
Bandgap (eV)	3.36	1.35	1.35
Electron affinity (eV)	4.0	4.07	3.7
Dielectric permittivity (relative)	66	23	23
CB effective density of states (cm^{-3})	1×10^{19}	1×10^{22}	1×10^{22}
VB effective density of states (cm^{-3})	1×10^{19}	1×10^{22}	1×10^{22}
Electron mobility (cm^2 V^{-1} s^{-1})	20	0.005	0.005
Hole mobility (cm^2 V^{-1} s^{-1})	20	0.005	0.005
Donor density (cm^{-3})	1×10^{18}		
Acceptor density (cm^{-3})		1×10^{16}	1×10^{16}
Electron thermal velocity (cm s^{-1})	1×10^{7}	1×10^{4}	1×10^{4}
Hole thermal velocity (cm s^{-1})	1×10^{7}	1×10^{4}	1×10^{4}
Defect type		Acceptor	Acceptor
Capture cross section electrons (cm^2)		1×10^{-15}	1×10^{-13}
Capture cross section holes (cm^2)		1×10^{-15}	1×10^{-13}
Energetic distribution		Single	Single
Energy level with respect to E_V (eV)		0.65	0.65
Trap density (cm^{-3})		5×10^{13}	5×10^{15}

Reference

1. Albaladejo-Siguan, M. *et al.* Interdot lead halide excess management in pbs quantum dot solar cells. *Adv. Energy Mater.* **12**, 2202994 (2022).

2. Heath, J. T., Cohen, J. D. & Shafarman, W. N. Bulk and metastable defects in $CuIn_{1-x}Ga_xSe_2$ thin films using drive-level capacitance profiling. *J. Appl. Phys.* **95**, 1000–1010 (2004).

3. Tauc, J. Optical properties and electronic structure of amorphous germanium. *Phys. Status Solidi B* **15**, 627–637 (1966).

4. Clifford, J. P. *et al.* Fast, sensitive and spectrally tuneable colloidal-quantum-dot photodetectors. *Nat. Nanotechnol.* **4**, 40–44 (2009).

5. Yoo, J., Jeong, S., Kim, S. & Je, J. H. A stretchable nanowire UV-Vis-NIR photodetector with high performance. *Adv. Mater.* **27**, 1712–1717 (2015).

6. Dong, R. *et al.* An ultraviolet-to-NIR broad spectral nanocomposite photodetector with gain. *Adv. Opt. Mater.* **2**, 549–554 (2014).

7. Pal, B. N. *et al.* High-sensitivity p-n junction photodiodes based on PbS nanocrystal quantum dots. *Adv. Funct. Mater.* **22**, 1741–1748 (2012).

8. Lee, J. W., Kim, D. Y. & So, F. Unraveling the gain mechanism in high performance solution-processed PbS infrared PIN photodiodes. *Adv. Funct. Mater.* **25**, 1233–1238 (2015).

9. Manders, J. R. *et al.* Low-noise multispectral photodetectors made from all solution-processed inorganic semiconductors. *Adv. Funct. Mater.* **24**, 7205–7210 (2014).

10. Wei, Y. *et al.* Hybrid organic/PbS quantum dot bilayer photodetector with low dark

current and high detectivity. *Adv. Funct. Mater.* **28**, 1706690 (2018).

11. Sliz, R. *et al.* Stable colloidal quantum dot inks enable inkjet-printed high-sensitivity infrared photodetectors. *ACS Nano* **13**, 11988–11995 (2019).

12. Vafaie, M. *et al.* Colloidal quantum dot photodetectors with 10-ns response time and 80% quantum efficiency at 1,550 nm. *Matter* **4**, 1042–1053 (2021).

13. Pina, J. M. *et al.* Quantum-size-effect tuning enables narrowband IR photodetection with low sunlight interference. *Nano Lett.* **22**, 6802–6807 (2022).

14. Lu, S. *et al.* High-performance colloidal quantum dot photodiodes via suppressing interface defects. *ACS Appl. Mater. Interfaces* **15**, 12061–12069 (2023).

15. Rauch, T. *et al.* Near-infrared imaging with quantum-dot-sensitized organic photodiodes. *Nat. Photonics* **3**, 332–336 (2009).

16. Goossens, S. *et al.* Broadband image sensor array based on graphene-CMOS integration. *Nat. Photonics* **11**, 366–371 (2017).

17. Liu, J. *et al.* A near-infrared colloidal quantum dot imager with monolithically integrated readout circuitry. *Nat. Electron.* **5**, 443–451 (2022).

18. Xu, Q. *et al.* Heterogeneous integration of colloidal quantum dot inks on silicon enables highly efficient and stable infrared photodetectors. *ACS Photonics* **9**, 2792–2801 (2022).
